# Overlapping Pathways: Autism Spectrum Disorder in Fragile X Carrier States and the Need for Greater Awareness

**DOI:** 10.7759/cureus.95948

**Published:** 2025-11-02

**Authors:** Maria V Fernandez, Alexandra Sirven, Brooke Pickles, Sheena Hamid, Aimee Nunez Selva

**Affiliations:** 1 Pediatric Medicine, Florida International University, Miami, USA; 2 Pediatric Medicine, American University of Antigua College of Medicine, St. John's, ATG

**Keywords:** autism spectrum disorder (asd), cgg repeats, fmr1, fragile x syndrome (fxs), fxand, gray zone, intermediate allele, neurodevelopmental disorder, premutation

## Abstract

Fragile X syndrome (FXS), caused by a full mutation (>200 CGG repeats) in the gene Fragile X messenger ribonucleoprotein 1 (FMR1), is the most common single-gene cause of autism spectrum disorder (ASD). Far less is known about the clinical significance of smaller FMR1 expansions. In this case series, we describe four male patients with ASD who carried the FMR1 intermediate (gray-zone) alleles (45-50 CGG; counts: 45, 49, 49, 50). All had early speech/language delay with ASD-consistent features (social-communication deficits, sensory sensitivities, and repetitive behaviors). Common comorbidities included attention-deficit/hyperactivity disorder (ADHD) features and sleep disturbance responsive to standard interventions. None of them met premutation (55-200 CGG) or full-mutation criteria. These observations align with emerging evidence that gray-zone alleles can be associated with neurodevelopmental phenotypes, including ASD traits, in a subset of carriers. Clinically, the series reinforces guidance to include FMR1 analysis in genetic evaluations for unexplained developmental delay/ASD and to provide counseling when intermediate results are identified. Awareness of a potential overlap between the FMR1 gray-zone states and ASD may aid comprehensive evaluation and family counseling. Larger, controlled studies, especially those incorporating AGG interruption analysis, are needed to quantify risk and clarify mechanisms underlying phenotypic variability.

## Introduction

Autism spectrum disorder (ASD) is characterized by persistent social-communication deficits and restricted, repetitive behaviors, with variable functional impact and frequent co-occurring conditions [1,2]. Among genetic etiologies, Fragile X syndrome (FXS) is the most prevalent inherited cause of intellectual disability and the most common single-gene cause of ASD; estimates suggest FXS accounts for ~1-6% of ASD cases [3-6]. Accordingly, professional guidance recommends genetic testing, including FMR1 analysis, during evaluation of children with unexplained developmental delay/ASD, particularly males [7].

FXS results from CGG expansion in the 5′ untranslated region of the gene Fragile X messenger ribonucleoprotein 1 (FMR1; Xq27.3). Full mutations (>200 CGG) typically cause promoter hypermethylation, transcriptional silencing, and loss of Fragile X mental retardation protein (FMRP), an RNA-binding protein essential for synaptic maturation and activity-dependent translation [3-6]. Perturbed synaptic plasticity, including altered metabotropic glutamate receptor (mGluR) signaling and gamma-aminobutyric acid or GABAergic tone, contributes to cognitive-behavioral phenotypes overlapping ASD [4-6].

Smaller expansions are categorized as premutation (55-200 CGG) and intermediate (gray-zone). Following the American College of Medical Genetics and Genomics (ACMG)-aligned clinical conventions, we define intermediate/gray-zone as 45-54 CGG (our cases: 45-50 CGG), acknowledging some laboratories use 41-54 CGG as “borderline” [3,8-12]. Repeat size can expand across generations, especially via maternal transmission, and AGG interruptions interspersed within the CGG tract modulate stability; and fewer AGGs increase expansion risk [8,9]. Premutation carriers often have elevated FMR1 mRNA with near-normal or modestly reduced FMRP; and RNA-mediated toxicity is implicated in Fragile X-Associated Tremor/Ataxia Syndrome and Fragile X-Associated Primary Ovarian Insufficiency (FXTAS/FXPOI) and in neurodevelopmental/psychiatric features grouped as Fragile X-associated neurodevelopmental disorder (FXAND) in some carriers [11,13-18]. By contrast, gray-zone carriers generally have normal FMRP; subtle RNA-level changes have been reported inconsistently, and clinical significance, particularly in the childhood, remains unresolved [3,12,13].

We present four male patients (three pediatric, one young adult) with ASD and FMR1 intermediate expansions (45-50 CGG) to highlight overlapping clinical features, emphasize appropriate testing and counseling, and underscore research gaps.

## Case presentation

Patient A

A five-year-old male patient with ASD had FMR1 49 CGG repeats (polymerase chain reaction (PCR) with methylation analysis at age three). Family history included a sibling with ASD reportedly carrying an FMR1 expansion below full mutation (range not provided). Early awake-only EEG showed a symmetric background without epileptiform activity. At age five, he demonstrated age-appropriate gross motor skills and general cognition with mild fine-motor/self-help delays (e.g., heel-to-toe walking, dressing). Expressive language delay persisted; he received speech therapy, occupational therapy (OT), and behavioral supports with good school engagement. Sleep fragmentation (with occasional enuresis) improved on nightly melatonin. A soft, musical grade 2/6 systolic murmur was considered likely innocent, and cardiology evaluation was arranged. Overall health was unremarkable.

Patient B

A two-year-old male patient with global developmental delay presented with late gross-motor milestones (walked at 18 months) and prominent communication/feeding difficulties (one- to two-word phrases; oromotor/sensory aversion to textures). Genetic testing identified FMR1 50 CGG repeats (PCR sizing; methylation consistent with non-full mutation). Maternal prenatal screening documented carrier status (exact repeat count unavailable). An older sister had absence seizures (carrier status unknown). At age two, he exhibited repetitive behaviors (lining up toys), sensory-seeking behavior, nighttime hyperactivity, and social-communication deficits consistent with ASD. Early-intervention services were initiated.

Patient C

An eight-year-old male patient with ASD and attention-deficit/hyperactivity disorder (ADHD) had FMR1 49 CGG repeats (PCR sizing). Multiple relatives reportedly had neurodevelopmental issues and/or Fragile X carrier status, though no immediate relatives had a full mutation. At age five, he showed hyperactivity, inattention, speech/language delay, and below-grade academic performance. He socialized well with family, was toilet-trained, fed himself (picky eater with texture aversions), and bathed with supervision but required prompts for more complex tasks and exhibited impulsive wandering. He received OT and speech therapy. By age six, he attended mainstream private school with a classroom aide and Registered Behavior Technician (RBT) support under an Individualized Education Program (IEP). Melatonin improved sleep maintenance. At age seven, he started stimulant therapy (mixed amphetamine salts) with improved attention/impulsivity; ongoing OT and music therapy aided regulation. He continued to have episodic tantrums and task avoidance but maintained passing grades with accommodations.

Patient D

A 22-year-old male patient with ASD had FMR1 45 CGG repeats (PCR sizing). Family history was negative for ASD or known Fragile X-associated conditions. Early childhood was notable for language delay, hyperactivity/impulsivity, and school behavior challenges. A pediatric neurologist diagnosed combined-type ADHD at age six. At age 11, he had a shower-associated episode of nausea/weakness with brief limb “jerking”; prior video-EEG showed generalized background slowing without epileptiform discharges, and antiseizure therapy was not initiated. Intensive speech/OT and school supports (IEP) improved communication and behavior. He aged out of pediatric care with persistent core ASD features requiring support; and no further seizure-like events were documented.

All the four cases and their key features are shown in Table 1.

**Table 1:** Case presentations and other key details DSM-5-TR: Diagnostic and Statistical Manual of Mental Disorders, Fifth Edition, Text Revision; ASD: autism spectrum disorder ; ADHD: attention-deficit/hyperactivity disorder; ST: speech therapy; OT: occupational therapy; RBT: Registered Behavior Technician; IEP: Individualized Education Program; vEEG: video-electroencephalography.

Case	Age/Sex	FMR1 CGG count	ASD diagnosis method & age	Comorbidities / notable history	Therapies / supports	Current status
A	5 y/M	49	Clinician DSM-5-TR; standardized tools variably used; age not documented	Sleep fragmentation with occasional enuresis; soft grade 2/6 systolic ‘innocent’ murmur	Speech therapy; occupational therapy; behavioral supports; nightly melatonin	Good school engagement; mild fine-motor/self-help delays; cardiology follow-up planned
B	2 y/M	50	Clinician DSM-5-TR at toddler age (documented at 2 y)	Global developmental delay; orosensory/feeding aversion; sibling with absence seizures	Early intervention (ST/OT/behavioral); sleep routines	Ongoing developmental therapies; ASD-consistent behaviors persist
C	8 y/M	49	Clinician DSM-5-TR; age not documented	ADHD (hyperactivity/inattention); texture aversion; impulsive wandering	OT; ST; melatonin; stimulant medication; IEP with aide and RBT; music therapy	Passing grades with accommodations; improved attention/impulsivity; episodic tantrums
D	22 y/M	45	Clinician DSM-5-TR in childhood; age not documented	Childhood ADHD; shower-associated presyncope with brief jerks; vEEG generalized slowing only	Intensive ST/OT and school supports in childhood; ongoing supports as adult	Persistent core ASD traits requiring support; no further seizure-like events

## Discussion

In this single-practice series, four male patients with ASD carried FMR1 intermediate (gray-zone) alleles (45-50 CGG). Shared features included early speech/language delay and ASD-consistent behaviors; several had ADHD symptoms and sleep disturbance responsive to standard measures. While causality cannot be inferred from a small, retrospective series, these observations are consistent with a potential association between gray-zone carrier states and ASD-related phenotypes in some individuals, extending prior adult-focused gray-zone observations [12,13] into pediatric and young-adult contexts.

Mechanistic context

Full mutations abrogate FMRP and perturb synaptic signaling, helping explain ASD overlap [3-6]. Premutation carriers (55-200 CGG) can exhibit RNA-mediated toxicity with modest FMRP effects, underpinning FXTAS/FXPOI and FXAND-type features (e.g., ADHD, anxiety, autism-spectrum traits) in subsets of carriers [11,14-18]. For gray-zone (45-54 CGG), FMRP is typically normal; subtle FMR1 transcript changes have been variably reported, and clinical significance, especially in the childhood, remains unsettled [3,12,13]. Our cases are hypothesis-generating within this framework.

Prior clinical data

In cohorts enriched for Fragile X families, a fraction of premutation carriers meet ASD criteria or show elevated ADHD/autism-spectrum traits versus controls [15-18]. Although subject to ascertainment bias, such findings support biological plausibility for spectrum-wide neurobehavioral effects below the full-mutation threshold. Adult gray-zone reports describe subtle neurologic/cognitive signs and, at a population level, associations with parkinsonism and earlier mortality [12,13]. Our pediatric/young-adult cases complement this literature by focusing specifically on gray-zone carriers with ASD.

Clinical implications

Current guidance recommends genetic evaluation for children with unexplained developmental delay/ASD, including FMR1 analysis, particularly in males and when family history is suggestive [7]. When FMR1 results fall in the intermediate range (45-54 CGG), genetic counseling is essential. Families should understand that (1) gray-zone status is not FXS; (2) intergenerational expansion risk exists, especially via maternal transmission, and depends partly on AGG interruption content [8-10]; and (3) some carriers may benefit from anticipatory guidance regarding co-occurring concerns (e.g., attention regulation, anxiety), while recognizing that FXAND is defined for premutation carriers and gray-zone associations remain uncertain [11,14-18]. Cascade testing can inform reproductive planning and identify at-risk relatives.

Limitations

This small, retrospective series from a single practice is subject to referral and documentation biases. Standardized research instruments were not uniformly recorded. 

## Conclusions

Four male patients with ASD in our practice carried FMR1 intermediate (gray-zone) expansions (45-50 CGG). Common features included early speech/language delay and ASD-consistent behaviors, and frequent comorbidities included ADHD features and sleep disturbance responsive to standard therapies. Clinically, these findings support including FMR1 analysis in evaluations for unexplained developmental delay/ASD and providing counseling when intermediate results are identified, both for management (e.g., attention/anxiety monitoring) and reproductive risk discussion. Scientifically, the series underscores the need for larger, controlled studies, integrating AGG analysis, to define the prevalence, penetrance, and mechanisms of ASD-related phenotypes among the gray-zone carriers.
